# EEG Classification of Different Imaginary Movements within the Same Limb

**DOI:** 10.1371/journal.pone.0121896

**Published:** 2015-04-01

**Authors:** Xinyi Yong, Carlo Menon

**Affiliations:** 1 School of Engineering Science, Simon Fraser University, Burnaby, British Columbia, Canada; University of Minnesota, UNITED STATES

## Abstract

The task of discriminating the motor imagery of different movements within the same limb using electroencephalography (EEG) signals is challenging because these imaginary movements have close spatial representations on the motor cortex area. There is, however, a pressing need to succeed in this task. The reason is that the ability to classify different same-limb imaginary movements could increase the number of control dimensions of a brain-computer interface (BCI). In this paper, we propose a 3-class BCI system that discriminates EEG signals corresponding to rest, imaginary grasp movements, and imaginary elbow movements. Besides, the differences between simple motor imagery and goal-oriented motor imagery in terms of their topographical distributions and classification accuracies are also being investigated. To the best of our knowledge, both problems have not been explored in the literature. Based on the EEG data recorded from 12 able-bodied individuals, we have demonstrated that same-limb motor imagery classification is possible. For the binary classification of imaginary grasp and elbow (goal-oriented) movements, the average accuracy achieved is 66.9%. For the 3-class problem of discriminating rest against imaginary grasp and elbow movements, the average classification accuracy achieved is 60.7%, which is greater than the random classification accuracy of 33.3%. Our results also show that goal-oriented imaginary elbow movements lead to a better classification performance compared to simple imaginary elbow movements. This proposed BCI system could potentially be used in controlling a robotic rehabilitation system, which can assist stroke patients in performing task-specific exercises.

## Introduction

A brain-computer interface (BCI) system translates human brain activity to commands that can operate a device, such as a computer [1]. Existing BCI systems have many applications. For example, a BCI allows a user to spell with a virtual keyboard [2, 3], to control an orthosis [4], a functional electrical stimulator (FES) [5], and to navigate the World Wide Web [6], with different degrees of success. In the early stage of BCI research, most BCI applications aimed to help people with limited mobility including those with amyotropic lateral sclerosis and spinal cord injury [7]. Recently, there is also an emerging interest in BCI with applications targeting stroke individuals. More specifically, investigations have been performed to evaluate the possibility of using BCIs for post-stroke rehabilitation to restore upper and lower limb functions [8, 9].

It is not straightforward to apply existing BCI systems to control devices such as a robotic exoskeleton. The main reason is that these systems have low dimensional control, i.e., they can only recognize a limited number of mental tasks as unique control commands. Motor imagery tasks such as left hand, right hand, and foot motor imagery are among the most frequently used in a BCI system [10]. Wolpaw and McFarland have shown that their participants were able to move a cursor with two-dimensional control (i.e., horizontal and vertical) on a computer screen after several sessions of training [11]. In this study, each dimension of cursor movement was controlled by the mu (8–12 Hz) or beta (18–26 Hz) rhythm, which was associated with left or right hand motor imagery. This strategy was then extended to three-dimensional cursor control (i.e., horizontal, vertical, and depth) in which the BCI was based on the changes in mu and/or beta rhythm during foot, left, and right hand motor imagery [12]. Scherer *et al.* have proposed a virtual keyboard controlled by a three-class BCI that discriminated the motor imagery of left hand, right hand, and foot [2]. Some studies employed intelligent control strategies to achieve multi-dimensional BCI control. For example, four-class BCIs have been developed, which allowed users to fly a virtual helicopter [13] and a robotic quadcopter in a three dimensional space [14]. The users would imagine moving/resting both hands to fly the helicopter forward/backward and imagine moving left/right hand to rotate the helicopter left/right. Doud *et al.* extended the work in [13] and introduced a six-class BCI. The third dimensional control of raising and lowering the helicopter was achieved by imagining moving the tongue and feet respectively [15]. They have demonstrated the ability of users to control the flight of a virtual helicopter with three dimensional control that can be independently adjusted in strength according to user preference.

While the classification of left hand, right hand, foot, and tongue motor imagery have been rather successful, the task of detecting the intention or discriminating the motor imagery of different movements within the same limb, on the other hand, is challenging. This is due to the fact that these motor tasks activate regions that have very close representations on the motor cortex area of the brain [16, 17]. To date, not many studies have addressed this problem. A summary of the studies that classify the motor imagery or the execution of different upper-extremity movements within the same limb is provided in Table 1.

**Table 1:** BCI studies that classify different same limb movements.

**Bibliography**	**# EEG**	**Real/MI**	**BCI Classes**	**Accuracy (%)**
Liao *et al.* [18]	128	Real	2-class: Different Pairs of Thumb, Index, Middle, Ring, Little Fingers	77.11% (11 Healthy)
Navarro *et al.* [19]	21	Real, MI	4-Class: Wrist Flexion, Extension, Pronation, Supination	35%, 34%, 35%, 32% (4 Healthy)
Vuckovic *et al.* [20]	64	Real, MI	2-Class: Different Pairs of wrist Flexion, Extension, Pronation, Supination	63%–82% (10 Healthy)
Ghani *et al.* [21]	64	Real	2-Class: different pairs of wrist Flexion, Extension, Pronation, Supination	61%–75% (3 Healthy)
Deng *et al.* [22]	163	Real (*Intention*)	2-Class: Shoulder Abduction, Elbow Flexion	89% (4 Healthy), 76% (1 Stroke)
Zhou *et al.* [23]	163	Real (*Intention*)	2-Class: Shoulder Abduction, Elbow Flexion	92% (4 Healthy), 75% (2 Stroke)
Chakraborti *et al.* [24]	2	Real	3-Class: Shoulder, Elbow, Finger	56%–93% (8 Healthy)

BCI studies that classify different same limb movements.

Liao *et al.* [18] have investigated the binary classification of the following ten different pairs of executed finger movements using 128-channel EEG signals: thumb vs index,; thumb vs middle; thumb vs ring; thumb vs little; index vs middle; index vs ring; index vs little; middle vs ring; middle vs little; and ring vs little finger. The average accuracy achieved in this study is 77.1% when power spectral changes are used as features and support vector machine is used as a classifier.

Three of the studies in Table 1 look into the decoding of different wrist movements. The classification of four different imaginary wrist movements namely wrist flexion, extension, pronation, and supination have been demonstrated in [19]. Unfortunately, the accuracies achieved are not satisfactory (approximately 35%). Vuckovic *et al.* [20] and Ghani *et al.* [21] also look into discriminating two different wrist movements using EEG signals. Their binary classification tasks include six combinations of different wrist movements: extension vs flexion; extension vs supination; extension vs pronation; flexion vs supination; flexion vs pronation; and supination vs pronation. The accuracies achieved in these studies are reasonably high (in the range of 60 to 80%). Vuckovic *et al.* [20] show that the best results were obtained when imaginary wrist extension was one of the classes being selected for classification. Ghani *et al.* [21], on the other hand, do not demonstrate any consistency in terms of the best classifiable type of movement.

Next, Deng *et al.* [22] and Zhou *et al.* [23] attempt to classify the intention of executing shoulder abduction and elbow flexion, which is used in a BCI system to overcome the abnormal coupling that exists between the shoulder abduction and elbow flexion following stroke. In these papers, the intention is defined as the time window of 1800 to 60ms prior to the onset of a voluntary shoulder or elbow torque. These two studies have demonstrated promising results, with accuracies above 70% for stroke patients and 80% for healthy volunteers. Finally, Chakraborti *et al.* [24] propose to use a multi-class BCI to control the motion and orientation of a robot. For each left and right hands, the execution of shoulder, elbow, and finger movements are classified using only 2-channel EEG signals. The classification of these same limb movements results in surprisingly high classification accuracies (i.e., in the range of 56%—93%).

In this paper, the research effort is focused in the classification of upper-limb movements within the same limb. We propose a 3-class BCI system that discriminates EEG signals corresponding to rest, imaginary grasp movements, and imaginary elbow movements. Motor imagery of grasp and elbow movements are chosen due to their potential use in controlling the robotic arm [25] developed in our lab and an FES. This rehabilitation system is designed to help stroke patients perform task-specific rehabilitation exercises and eventually improve their upper-extremity functions. The three classification tasks employed in this study are different from those listed in Table 1. Even though Chakraborti *et al.* [24] also look into the classification of elbow and finger movements, but their work focuses on real movements and resting states are not considered in their study. In contrast, both imaginary movements and a rest state are included in our classification problem. In the present study, we also investigate the differences between simple motor imagery and goal-oriented motor imagery in terms of their topographical distributions and classification accuracies.

To the best of our knowledge, the classification combination employed in this study as well as the difference between simple and goal-oriented motor imagery have not been explored in the BCI literature. In addition, all BCIs designed for stroke rehabilitation only classify two classes (left vs right motor imagery or mostly rest vs motor imagery), as shown in Table 2. A 3-class BCI system for stroke rehabilitation has some advantages over the state-of-the-art 2-class BCIs designed for stroke rehabilitation. First, it has an additional dimension to operate a robotic system when performing task-specific exercises. For example, the user can imagine elbow movements to move the robotic device close to a cup, and then imagine grasp movements to activate the FES, which in turn close the user’s fingers to grab the cup. Such control is more intuitive than that derived from a BCI system that identifies the motor imagery of different limbs (i.e., left/right hand and foot). The second advantage of the 3-class BCI system is that the users can perform mental practice on two different joint movements using the same device. Studies have shown that a rehabilitation program that includes mental practice can help improve the use and function of the affected arm of a stroke patient [26, 27].

**Table 2:** BCI studies in stroke rehabilitation, focusing on upper-extremity rehabilitation and EEG was used as a modality to measure brain activities.

**Bibliography**	**Feedback**	**BCI Classes**
Prasad *et al.* [28]	BCI + Visual	2-Class: MI Left vs MI Right (Arm/Hand)
Ortner *et al.* [29]	BCI + Visual	2-Class: MI Left vs MI Right (Hand)
Kaiser *et al.* [30]	BCI + Visual	2-Class: MI/AT (Grasp) vs Rest
Daly *et al.* [31]	BCI + Visual + FES	2-Class: MI/AT (Finger Extension) vs Relax
Tam *et al.* [32], Meng *et al.* [33]	BCI + Visual + FES	2-Class: MI (Wrist) vs Rest
Young *et al.* [34]	BCI + Visual + FES + TDU	2-Class: AT (Open + Close Hand) vs Rest
Tan *et al.* [35]	BCI + Visual + NMES	2-Class: MI (Hand) vs Rest
Ang *et al.* [36–38]	BCI + Visual + Robot	2-Class: MI (Hand) vs Rest
Ang *et al.* [39]	BCI + Visual + Robot	2-Class: MI (Grasp) vs Rest
Rodriguez *et al.* [40, 41]	BCI + Visual + Robot	2-Class: MI (Elbow Flexion) vs Rest;
		2-Class: MI (Elbow Extension) vs Rest
Buch *et al.* [42], Broetz *et al.* [43]	BCI + Visual + Orthosis	2-Class: MI/AT (Grasp) vs Rest (Open)
Shindo *et al.* [44]	BCI + Visual + Orthosis	2-Class: MI (Open Hand) vs Rest
Ramos-Murguialday *et al.* [45]	BCI + Orthosis	2-Class: AT (Reach & Grasp) vs Rest
Frisoli *et al.* [46]	BCI + Arm Exoskeleton + Kinect + Eye-Tracker	2-Class: MI (Right Arm) vs Rest
Cincotti *et al.* [47]	BCI + Visual / BCI + EMG + FES	2-Class: MI/AT (Grasp/Finger Extension) vs Rest

MI: Motor Imagery; AT: Attempted Movement; FES: Functional Electrical Stimulation; TDU: Tongue Stimulation;

NMES: Neuromuscular Electrical Stimulation.

BCI studies in stroke rehabilitation, focusing on upper-extremity rehabilitation and EEG was used as a modality to measure brain activities.

In the following section, the experimental procedures as well as the feature extraction and machine learning algorithms are described. Results are presented in Section 3 and Section 4 is dedicated to discussion and conclusion.

## Experimental Procedure

### EEG Recording

All of the methods within this study were in compliance with the declaration of Helsinki and were approved by the Simon Fraser University (SFU) Office of Research Ethics (#2012s0527). We recruited twelve able-bodied individuals for this study. Participants gave a written consent before participating in the experiment. Each individual was seated comfortably in front of a computer monitor. The computer provided a simple Graphical User Interface (GUI) that displays commands or cues to the participant.

**Fig 1:**
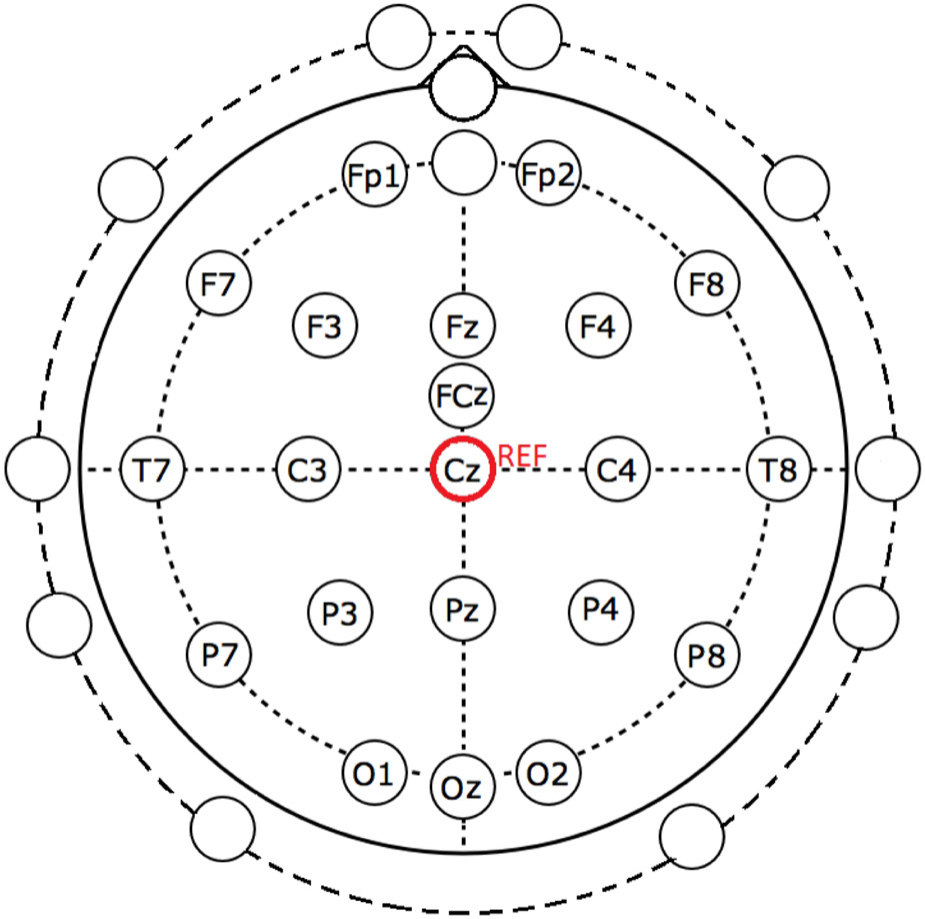
The EEG electrode positions employed in this study. The labeled electrodes were used in our BCI system. The remaining unlabeled electrodes, on the other hand, were not considered in this study. All these electrodes were referred to the vertex (Cz position).

A 32-channel EGI’s Geodesic sensor net was applied on the participant’s head [48]. The locations of all the electrodes are shown in Fig. 1. The labeled electrodes were those we employed for our BCI system. The remaining unlabeled electrodes, on the other hand, were not considered in this study because they were very close to sources that generate muscle activities or artifacts. All these electrodes were referred to the vertex (Cz position in Fig. 1) of the participant. The EEG signals were amplified and sampled at 1000 Hz using a Geodesic Net Amps 400 series amplifier [49]. Throughout the experiment, the electrode impedance was maintained below 50 kΩ.

### Experimental Procedures

**Fig 2:**
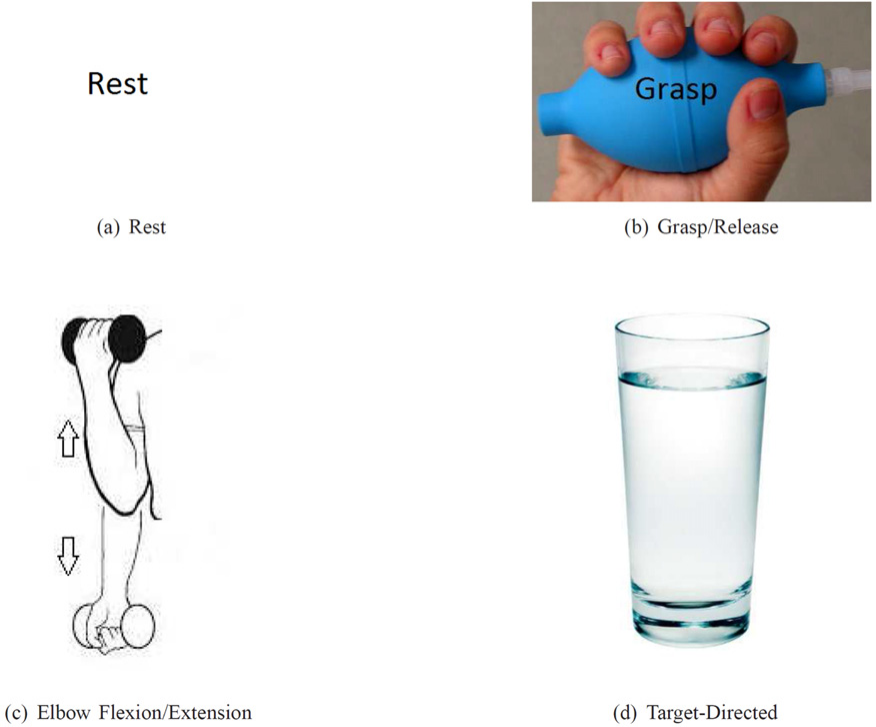
Visual cues presented during the experiments. (a) REST: rest and relax; (b) MI-GRASP: imagine opening and closing the fingers; (c) MI-ELBOW: imagine moving the forearm up and down; (d) MI-ELBOW-GOAL: imagine reaching out for the glass of water displayed on the computer monitor and bringing it back.

Each experiment for each participant lasted for approximately 1.5 hours. The experiment consisted of four sessions. Each session lasted 12 minutes. The participant was asked to perform different repetitive tasks according to the visual cues displayed on the computer monitor. Four different visual cues (see Fig. 2) were presented in a random order to the participant. They are listed as follows:

1. Rest (REST): rest and relax [Fig. 2(a)]
2. Motor imagery of grasp (MI-GRASP): imagine opening and closing all the fingers to grab an object [Fig. 2(b)]
3. Motor imagery of elbow flexion and extension (MI-ELBOW): imagine moving the forearm up and down [Fig. 2(c)]
4. Goal-directed motor imagery of elbow flexion and extension (MI-ELBOW-GOAL): imagine reaching out for the glass of water displayed and bringing it back [Fig. 2(d)]

There is a clear distinction between MI-ELBOW and MI-ELBOW-GOAL. MI-ELBOW involves only simple repetitive elbow flexion and extension. MI-ELBOW-GOAL on the other hand is a goal-oriented action, i.e., a visible goal (a glass of water) is present. MI-ELBOW-GOAL was included in this study to investigate the effect of goals or targets on EEG activity and consequently on the classification accuracy of the multi-class BCI system proposed.

**Fig 3:**
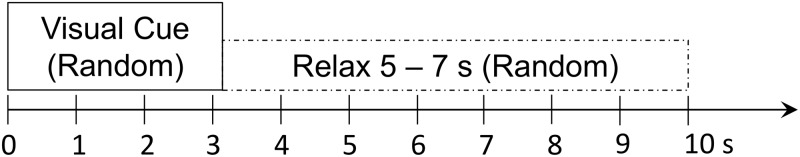
Experimental paradigm. At 0 s, a visual cue is randomly selected and presented. After 3 s, a blank screen appears for 5 −7 s before another visual cue is presented. During this period of time, the participant is requested to rest.

Each session consisted of 20 trials for each tasks. Each trial lasted from 8 to 10 s (see Fig. 3). Each visual cue was randomly selected and displayed on the screen for 3 s, indicating which task to perform. The participant was asked to perform each designated task for 3 s, followed by 5 to 7 s of rest. Throughout the experiment, the participant could take a break whenever needed.

## Feature Extraction and Classification

The EEG data collected from each experiment contained a mixture of four different mental states: REST, MI-GRASP, MI-ELBOW, and MI-ELBOW-GOAL. In this paper, we first looked into the binary classification of the following combinations:

1. REST vs MI-GRASP
2. REST vs MI-ELBOW
3. REST vs MI-ELBOW-GOAL
4. MI-GRASP vs MI-ELBOW
5. MI-GRASP vs MI-ELBOW-GOAL

Next, the classification of the following three classes were performed:

1. REST vs MI-GRASP vs MI-ELBOW
2. REST vs MI-GRASP vs MI-ELBOW-GOAL

The EEG data were processed by a signal processing unit that performs signal preprocessing, feature extraction, and classification operations. The relevant features were extracted and translated to useful control signals that could be employed to control one or more devices. In a three-class classification problem, the output of the classifier had one of the three discrete states ‘0’, ‘1’, or ‘2’ and was not a continuous function. The logical states ‘1’ and ‘2’ indicated the user’s intention to activate a device (e.g. a robotic arm or an FES). The logical states ‘0’, on the other hand, implied that the user did not intend to activate the system.

In this study, an open-source MATLAB toolbox, BCILAB, was utilized to process the EEG data [50]. In the following subsections, details about the data preprocessing, feature extraction, and classification algorithms are given.

### Data Preprocessing

The EEG data were downsampled to 250 Hz and then band-pass filtered to the 6–35 Hz frequency band. This frequency band encompasses the mu and beta rhythms which have been reported to desynchronize during motor imagery [51]. The band power changes of the mu and beta rhythms have been successfully used in BCI systems to classify EEG signals related to motor imagery [52–54]. Also, by band-pass filtering the data, ocular artifacts caused by the low frequency components of the EEG data were minimized.

### Feature Extraction

EEG epochs from 1 to 3 s after a visual cue were segmented. Then, features were extracted from each segment. The following feature extraction methods, which are widely used in BCI research, were employed:

1. Common Spatial Patterns (CSP) [53]
2. Filter-Bank Common Spatial Patterns (FBCSP) [55]
3. Logarithmic Band Power (BP) [10]

The frequency window used and the feature dimension for each method are presented in Table 3.

**Table 3:** Frequency window, time segment, and feature dimension for each feature extraction method.

**Method**	**Frequency Window**	**Time Segment**	**Feature Dimension**
CSP	7–30 Hz	1–3 s	6
FBCSP	7–15 Hz, 15–25 Hz, 25–30 Hz	1–3 s	18
BP	7–30 Hz	1–3 s	20

Feature extraction algorithms used in this study.

CSP has been widely used in BCI research to extract features from EEG signals. This algorithm can effectively extract discriminatory information from two classes of EEG signals [53]. The algorithm finds the directions where the EEG signals should be projected onto so that the differences between any two classes of EEG signals are maximized (i.e. the variance of one class is maximized while at the same time, the variance of the other class is minimized) [52]. These directions are provided by a weight matrix in which its rows give the weights of the EEG channels.

Here, the formulation of the CSP algorithm for a 2-class problem is described. This same formulation of the 2-class CSP algorithm was also used when classifying the three classes of EEG signals in this study as only binary classifiers were trained. More specifically, for a 3-class problem, three different binary classifiers were trained and a voting scheme was employed to determine the class label. More details about the voting scheme is provided in the next subsection.

Given two classes of EEG signals: Class 1 and Class 2, the CSP algorithm finds a spatial filter such that the signals can be projected into a 1-dimensional space where one class of signals is maximally scattered and the other is minimally scattered. High variance of the signals indicates strong rhythms whereas low variance indicates attenuated rhythms [52]. Let *S* = {*S*_1_,*S*_2_,…,*S*_*M*_} where *S*_*i*_ ∈ ℝ^*N*_*c*_×*N*^ denotes the filtered *i*-th trial EEG signal, *M* the number of EEG trials, *N*_*c*_ the number of EEG channels, and *N* the number of samples in the signal. The optimization problem is expressed as:

$$\begin{array}{c} \min_{w}\sum_{i\in {𝒞}_{1}}\mathrm{var}\left(w^{T}S_{i}\right)\\ s.t.\sum_{i=1}^{2M}\mathrm{var}\left(w^{T}S_{i}\right)=1 \end{array}$$ (1)

where 𝒞_1_ represents all Class 1 EEG trials and $w\in {\mathbb{R}}^{N_{c}}$ is the unknown weight vector of the spatial filter. In this study, the CSP features selected for classification were the log-variance of the EEG signals projected using six different spatial filters. These spatial filters were a) the three most important spatial filters that explain the largest variance of Class 1 and the smallest variance of Class 2 and b) the three most important spatial filters that explain the largest variance of Class 2 and the smallest variance of Class 1.

FBCSP is an extension of the CSP algorithm [55]. First, a filter bank is used to bandpass filter the EEG signals. Then, for each filtered EEG band, spatial filters are found using the CSP algorithm discussed earlier. In this study, three filtered EEG bands were generated: 7–15 Hz, 15–25 Hz, and 25–30 Hz. The FBCSP features selected for classification were the log-variance of each of the filtered EEG band projected using six different spatial filters.

The third method, logarithmic band power (BP) is a simpler method. The features used for classification were the log-variance of the bandpass filtered EEG signals from every channel.

### Classification

Three classifiers were used to classify the three-class data:

1. Linear Discriminant Analysis (LDA) [56]
2. Logistic Regression (LR) using a fast Bayesian method [57]
3. Support Vector Machine (SVM) with a radial basis function (RBF) kernel [58]

For the SVM with an RBF kernel (*K*(*x*,*y*) = *e* ^−*γ*∣∣*x*−*y*∣∣^2^^), two parameters were optimized: a) the kernel parameter gamma *γ* and b) the penalty weight *c*, which acts like a regularization parameter that controls the misclassification rate of the training data. The optimal parameters were obtained from a grid search with *c* ranging from 2^−5^ to 2^15^ and *γ* ranging from 2^−15^ to 2^3^[59].

To apply these three machine learning algorithms to a multi-class problem, an one-vs-one voting strategy was employed. In the one-vs-one voting scheme, *K*(*K*−1)/2 binary classifiers for a *K*-way multi-class problem are trained. During testing, all the binary classifiers are applied to an unseen sample and the class that receives the highest number of votes wins [58]. In our case, *K* = 3 and for each 3-class classification problem, 3 binary classifiers were set up.

Next, to evaluate the performance of the 3-class BCI system, the 10 × 10 cross-validation method was employed [58]. The data set was randomized and divided into ten folds. Nine of the folds were used to set up the classifier and the remaining one fold was used to test the classifier. This procedure was repeated for ten times. Then, the average cross-validation classification accuracy was computed and used as a performance metric.

In this study, nine combinations of different feature extraction and classification algorithms listed above were used to discriminate the different classes of EEG signals. For each participant, the highest cross-validation accuracy obtained from one of the nine algorithms mentioned earlier was reported.

## Results

### ERD/ERS Analysis

It is known that motor imagery, preparation for movement, or movement is usually accompanied by a decrease in the mu and beta rhythms over the sensorimotor cortex area especially the contra-lateral region [51, 60]. This decrease is also known as event-related desynchronization (ERD). A recent EEG and fMRI study suggested that the degree of this decrease might be quantitatively associated with an increase in neuronal activity [60]. Besides ERD, an increase in the beta rhythm also occurs after a motor imagery or a movement is executed. This increase is known as event-related synchronization (ERS). In this section, the average time course for the ERD and ERS obtained from the contra-lateral C3 location of all participants are presented.

**Fig 4:**
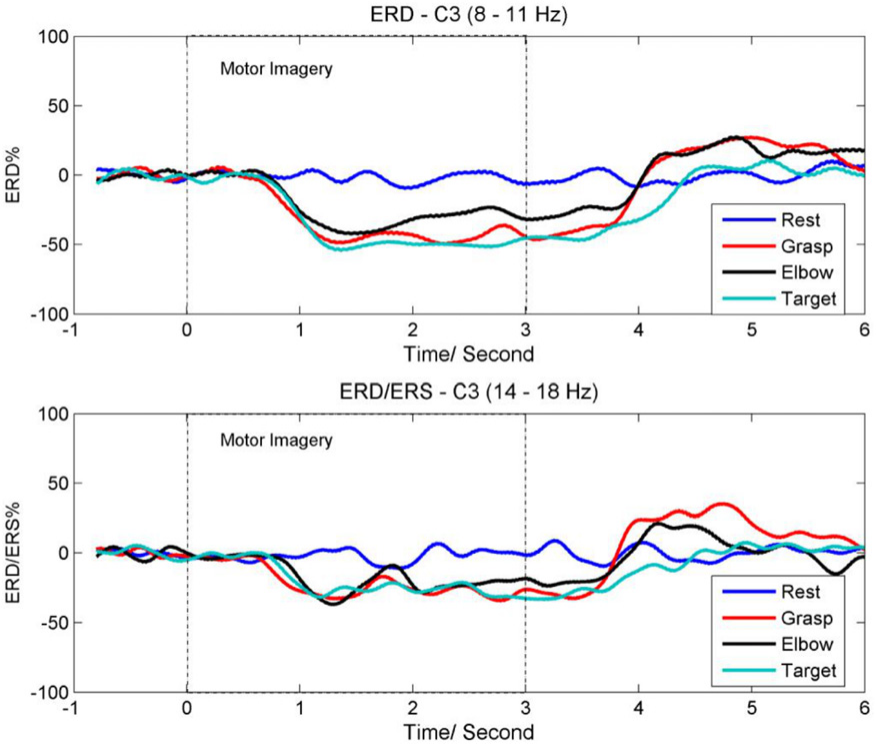
The average time course for ERD and ERS. (a) ERD time course for the mu rhythm (8–11 Hz) at the C3 location; (b) ERD/ERS time course for the beta (14–18 Hz) rhythm at the C3 location.

Fig. 4(a) shows the ERD time course for the mu rhythm (8–11 Hz) at the C3 location. Visual cues were prompted on the computer screen from time 0s to 3s. The ERD time course was obtained by averaging the power changes of the mu rhythm across all trials and all participants. As shown in the figure, the power of the mu rhythm is attenuated approximately 0.7s after the onset of MI-GRASP, MI-ELBOW, and MI-ELBOW-GOAL. Also, MI-ELBOW-GOAL produces greater ERD as compared to MI-ELBOW. As it takes time for the participants to see the cue, decide which task to perform and then react, the attenuation of the mu rhythm does not start at the onset of the motor imagery tasks. About 1s after the participants stop imagining, the ERD recovers to the rest and baseline level. The mu rhythm for MI-ELBOW-GOAL takes an additional 400ms to recover to the baseline level.

Next, Fig. 4(b) shows the ERD/ERS time course for the beta (14–18 Hz) rhythm at the C3 location. The time course was obtained by averaging the power changes of the beta rhythm across all trials and all participants. As shown in the figure, the beta rhythm also displays an attenuation in its power after the onset of MI-GRASP, MI-ELBOW, and MI-ELBOW-GOAL. In addition, ERS or a rebound in the beta rhythm is observed after the participants have completed the motor imagery tasks of MI-GRASP and MI-ELBOW. However, no ERS is observed in the case of MI-ELBOW-GOAL.

**Fig 5:**
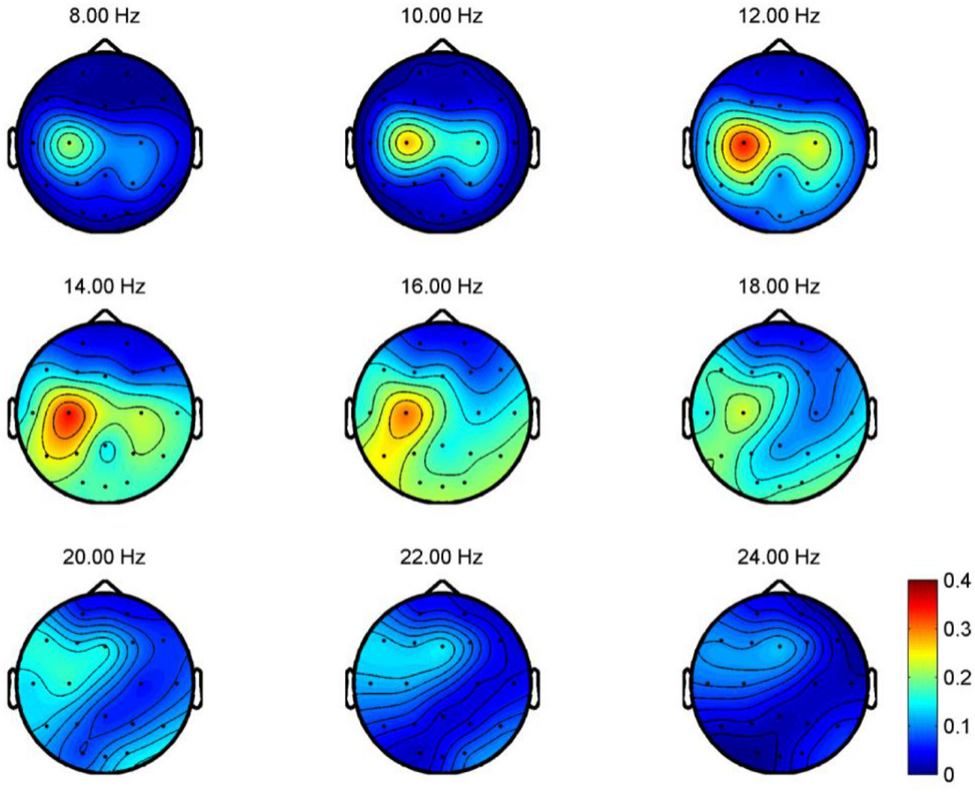
R^2^ values for REST vs MI-GRASP for P06. *R* ^2^ measures the difference between two classes. In this figure, the *R* ^2^ values for frequency bands ranging from 8 to 24 Hz at each electrode locations are computed for all participants. Large *R* ^2^ values are observed at electrode locations near the contra-lateral motor cortex area.

To illustrate the topographical distribution on the scalp of the difference between rest and imaginary grasp movements, the *R* ^2^ values for frequency bands ranging from 8 to 24 Hz at each electrode locations are computed for all participants. *R* ^2^ measures the difference between two classes, i.e., the proportion of the single-trial variance that is due to the task [1]. The topographical map of one of the participants (P06), which demonstrates prominent scalp difference between rest and imaginary grasp movements, is shown in Fig. 5. In this figure, large *R* ^2^ values are observed at electrode locations near the contra-lateral motor cortex area. Such prominent differences occur as a result of the ERD of the mu and beta rhythms when MI tasks are executed.

### Frequency and Topographical Analysis for Different MIs

**Fig 6:**
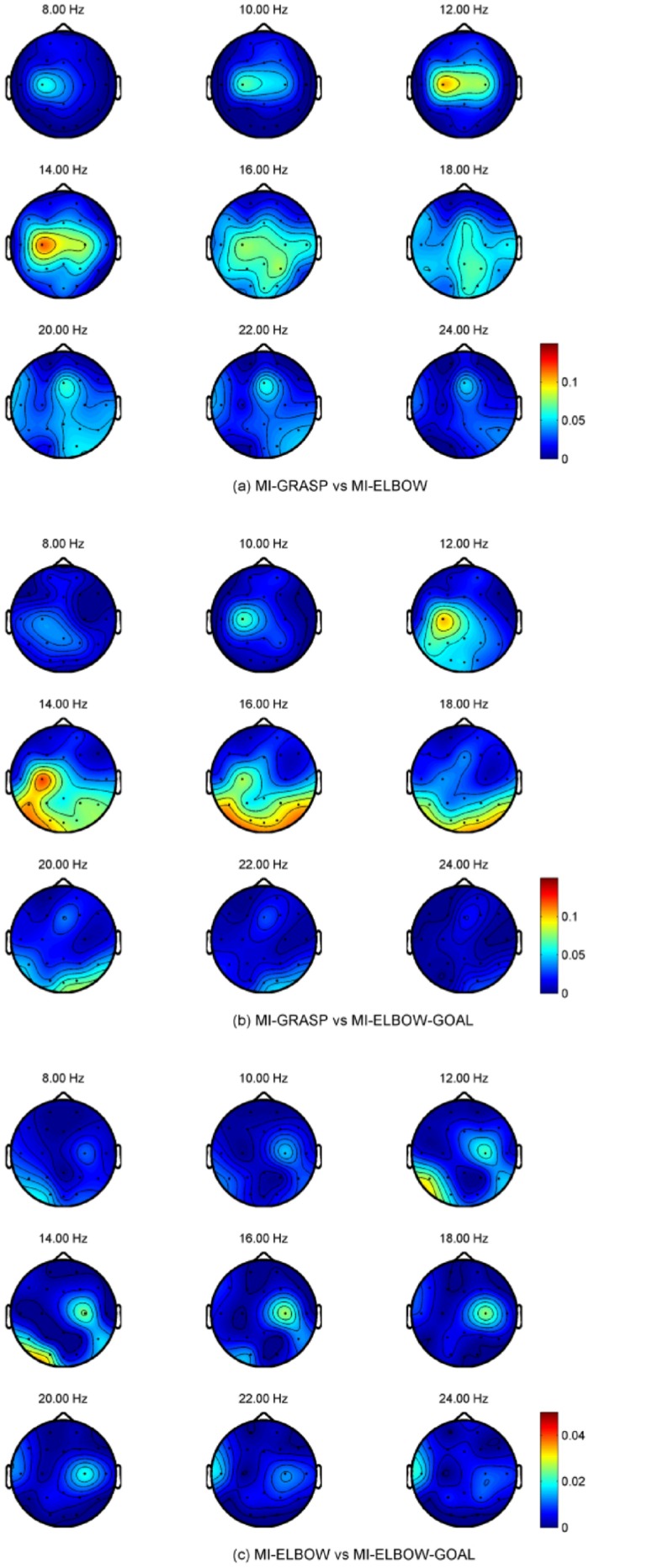
R^2^ values for P06 when different MI tasks were performed.

**Fig 7:**
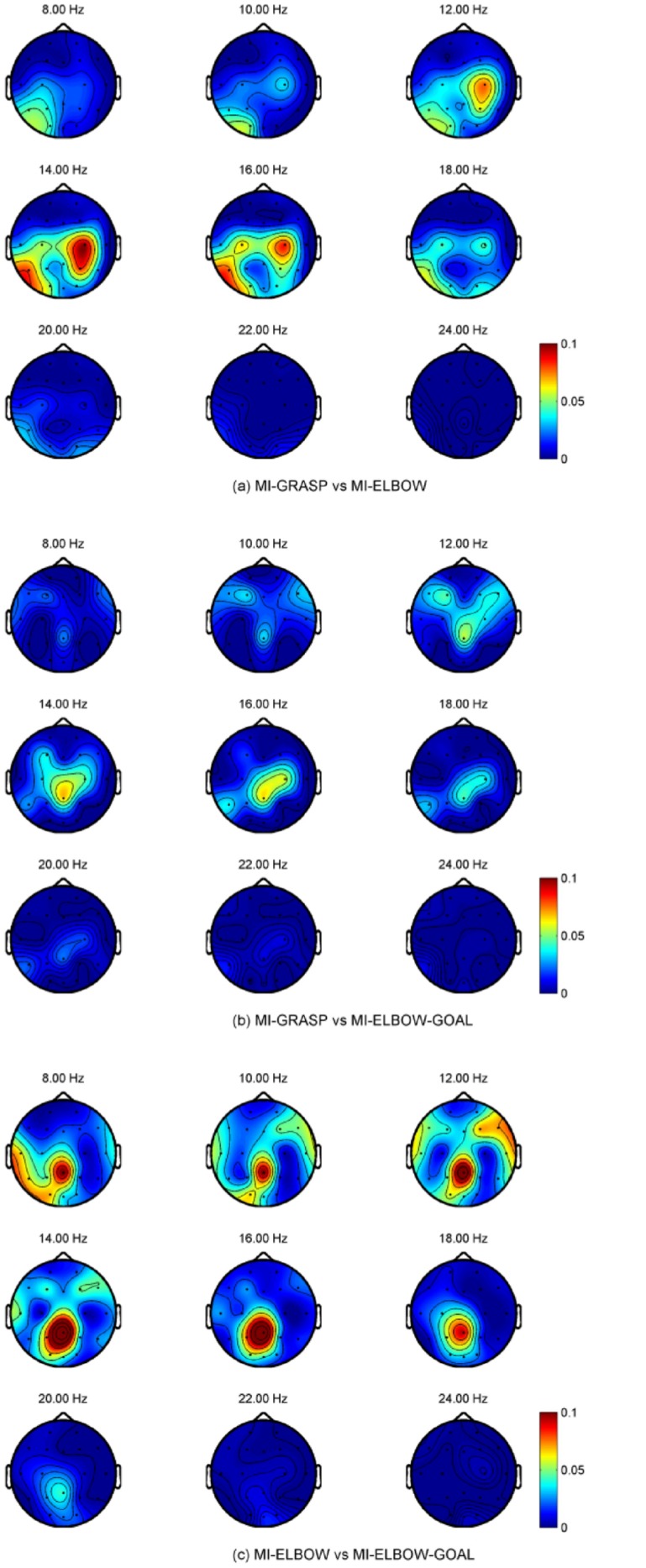
R^2^ values for P07 when different MI tasks were performed.

We are also interested in the topographical distribution on the scalp for different motor imagery tasks measured by *R* ^2^ values. Unfortunately, the topographical difference is subject-specific and no consistent patterns can be observed. Examples are taken from participants PO6 and P07 to reflect this difference and their topographical maps are presented in Fig. 6 and Fig. 7 respectively. For the case of MI-GRASP vs MI-ELBOW, larger difference is observed in the contra-lateral of the motor cortex in participant P06 but in the ipsi-lateral of the motor cortex in participant P07. When comparing MI-ELBOW against MI-ELBOW-GOAL, larger *R* ^2^ values are observed in the ipsi-lateral of the motor cortex in participant P06 but in the Pz region for participant P07. Finally, for MI-GRASP vs MI-ELBOW-GOAL, the difference in terms of the *R* ^2^ values is the greatest in the contra-lateral of the motor cortex and the visual cortex (for the frequency range of 14–18 Hz) in participant P06. Participant P07, on the other hand, has the greatest difference in the Pz region and the ipsi-lateral of the motor cortex.

### 2-Class Classification Results

Table 4 shows the classification accuracy achieved for the binary classification of REST against another type of motor imagery (i.e., MI-GRASP, MI-ELBOW, or MI-ELBOW-GOAL) using EEG signals. For each participant, the reported classification accuracy is in fact the highest accuracy obtained from the different combinations of feature extraction and classification algorithms described in the previous section. The results obtained are consistent with those achieved in the literature [8, 9]. The best results are achieved for the binary classification of REST VS MI-GRASP (80.5%).

**Table 4:** Binary classification accuracies for REST and a MI task.

**Participant**	**2-Class Accuracy/%**		
	REST vs MI-GRASP	REST vs MI-ELBOW	REST vs MI-ELBOW-GOAL
P01	85.0±8.9	83.4±8.3	81.8±9.2
P02	88.4±7.6	87.9±7.2	86.7±8.1
P03	90.1±6.7	86.7±7.1	89.5±7.3
P04	74.6±10.3	74.2±9.9	70.1±11.5
P05	85.9±8.6	82.8±10.0	78.1±10.7
P06	84.2±7.8	75.7±10.0	71.1±11.1
P07	81.1±10.2	69.6±11.3	76.4±9.9
P08	68.4±10.3	62.6±12.7	68.3±11.4
P09	83.6±9.0	73.4±10.4	82.2±8.9
P10	89.3±7.4	91.0±6.2	85.2±8.6
P11	63.1±12.9	59.0±9.2	65.0±12.4
P12	72.3±12.0	67.8±12.1	64.5±11.7
**Mean**	**80.5±8.8**	**75.1±11.3**	**76.6±8.7**

Binary classification accuracies for REST and a MI task.

To compare the results of the three different binary classifiers, one-way ANOVA is used since the data are normally distributed as assessed by Shapiro-Wilk Test (*p* > 0.05) [61]. Besides, the variances of the data are homegeneous according to the Levene’s test for variance homogeniety (*p* > 0.05). The analysis shows that the means of the performance of the BCI for different binary combinations are not statistically significant (*p* > 0.05).

Table 5 presents the classification accuracy achieved for two different binary classifiers: MI-GRASP vs MI-ELBOW and MI-GRASP vs MI-ELBOW-GOAL. The data are normally distributed as assessed by Shapiro-Wilk Test (*p* > 0.05). Thus, the paired t-test [61] is used to test the statistical significance of the results. The analysis shows that the means of the performance of the BCI for different MI combinations are significantly different at a significance level of 0.05.

**Table 5:** Binary classification accuracies for two different MI tasks.

**Participant**	**2-Class Accuracy/%**	
	MI-GRASP vs MI-ELBOW	MI-GRASP vs MI-ELBOW-GOAL
P01	59.9±12.2	58.8±11.8
P02	58.0±13.4	74.3±10.1
P03	60.9±11.4	59.8±12.1
P04	60.0±12.5	70.1±11.5
P05	58.3±11.7	63.3±10.8
P06	68.1±11.8	72.3±10.4
P07	69.2±11.6	80.6±9.9
P08	58.0±11.2	62.6±10.7
P09	56.9±12.0	70.2±12.2
P10	69.0±11.9	69.8±10.3
P11	50.0±12.7	56.1±11.8
P12	62.6±11.6	64.6±12.2
**Mean**	**60.9±5.6**	**66.9±7.2**

Binary classification accuracies for two different MI tasks.

Table 5 also shows that higher classification accuracies are achieved for the combination that involves MI-ELBOW-GOAL except participant P01 and P03, whose classification rates for both cases are about the same. Large accuracy gains when using the goal-oriented strategy are observed in participants P02, P04, P07, and P09 where the increment ranges from 10.1 to 16.3%.

### 3-Class Classification Results

Table 6 shows the performance achieved by the BCI when classifying three classes of mental tasks, i.e., REST vs MI-GRASP vs (MI-ELBOW or MI-ELBOW-GOAL) using EEG signals. The data are normally distributed as assessed by Shapiro-Wilk Test (*p* > 0.05). The paired t-test is used to compare the accuracies for the non goal-oriented and goal-oriented 3-class classification problems. The analysis shows that the means of the performance of the BCI for different combinations are statistically significant (*p* < 0.05).

**Table 6:** Performance of the 3-Class BCI.

**Participant**	**3-Class Accuracy/%**	
	Simple	Goal-Oriented
P01	58.2±9.6	59.4±10.0
P02	62.8±9.3	72.9±10.0
P03	65.9±8.9	66.2±9.1
P04	53.1±8.3	59.1±8.9
P05	58.8±9.0	59.8±9.8
P06	61.1±9.1	61.2±8.6
P07	56.3±8.8	67.4±8.9
P08	47.6±9.6	50.8±9.6
P09	52.5±9.7	66.1±9.8
P10	70.3±9.1	69.4±8.9
P11	40.1±9.7	45.3±11.8
P12	48.3±8.8	50.4±11.0
**Mean**	**56.2±8.5**	**60.7±8.4**

Performance of the 3-Class BCI.

As shown in the table, higher classification accuracies are achieved for the combination that involve MI-ELBOW-GOAL except participant P6 and P10, whose classification rates for both cases are about the same. Large accuracy gains when using the goal-oriented strategy are observed in participants P02, P07, and P09 where the increment ranges from 10.1 to 13.6%.

## Discussion

In this paper, we first look into the binary classification of different imaginary movements such as MI-GRASP, MI-ELBOW, and MI-ELBOW-GOAL. Then, the possibility of designing a 3-class BCI that discriminates rest, imaginary grasp movements, and imaginary elbow movements is investigated. This paper also investigates whether goal-oriented motor imagery outperforms non-goal-oriented motor imagery when classifying the task against other imaginary task and/or rest. In the following subsections, more details about our claims and results are provided.

### Multi-Class Classification

The main aim of the present study is to investigate the possibility of detecting the motor imagery (MI) of different joint movements within the same limb as well as detecting MI from a rest state. To the best of our knowledge, this is one of the first study to distinguish both the imaginary grasp movements and imaginary elbow movements from resting states using EEG signals. Besides, we also investigate whether a goal-oriented motor imagery reaching task using elbow (a functional movement) produces EEG features that are more prominent when compared to a non-goal oriented motor imagery elbow flexion and extension.

From Table 4, it has been demonstrated that the binary classifiers achieve an average classification accuracy of 80.5%, 75.1%, and 76.6% for REST vs MI-GRASP, REST vs MI-ELBOW, and REST vs MI-ELBOW-GOAL respectively. These results are consistent with the performances reported in the literature [8, 9]. The classification of different MI from the same limb is more challenging. The best classification pair is MI-GRASP vs MI-ELBOW-GOAL with a classification accuracy of 66.9% (Table 5). This performance is significantly better than the binary classification pair of MI-GRASP vs MI-ELBOW.

The binary classification problem is then extended to a multi-class classification problem. For this 3-class classification problem, an average accuracy of 60.7% is achieved and all the participants have accuracies well above the random classification level of 33.3%. As expected, the classification accuracies are lower than those achieved by the binary classifiers. The deterioration in the accuracy is caused by the difficulty in discriminating MI-GRASP from MI-ELBOW or MI-ELBOW-GOAL. It is challenging to discriminate the motor imagery of different movements within the same limb because these motor tasks activate regions that have very close representations on the motor cortex area of the brain [16, 17]. As the number of electrodes placed around the motor cortex area is sparse, we could expect better performance when denser electrodes are placed over the scalp. Other approaches that can potentially improve the classification performance of the BCI system include more BCI training and the use of online feedback. Besides, a hybrid BCI that combines EMG and EEG could also potentially improve the efficiency and practicality of the system.

### Simple vs Goal-Oriented Motor Imagery

Based on the ERD/ERS as well as frequency and topographical analysis using *R* ^2^ values, we found differences between MI-GRASP and MI-ELBOW or MI-ELBOW-GOAL especially at the motor cortex area of the brain. Such differences are not consistent across all the participants. For example, for participant P07, the *R* ^2^ values for MI-ELBOW vs MI-ELBOW-GOAL are prominent in the posterior parietal cortex area, which is consistent with [62] in which the imagined goal reaching task has been shown to activate areas that are posterior and medial in the parietal cortex area. Moreover, evidence also shows that the parietal cortex is involved in movement planning [63, 64]. In participant P07, visual areas were also activated probably because the participant performed visual imagery during the MI-ELBOW-GOAL tasks. The activations were stronger in the left hemisphere, as the participant in right-handed. For participant P06, no significant difference in the *R* ^2^ values for MI-ELBOW vs MI-ELBOW-GOAL is observed. Hence, there was no difference between the classification performance when a simple or a goal-oriented imaginary elbow movement was involved.

For both binary classification and 3-class classification as shown in Table 5 and Table 6 respectively, higher classification accuracies are achieved for the combinations that involve MI-ELBOW-GOAL except two participants. From these tables, large accuracy gains when using the goal-oriented strategy are observed in participants P02, P07, and P09. For the binary classification of MI-GRASP and MI-ELBOW-GOAL, the gain is 16.3% for participant P02. The goal-oriented version of the motor imagery, MI-ELBOW-GOAL leads to a significantly higher accuracy probably because an goal-oriented action activated more regions of the brain. It could also be due to the fact that the participants were able to focus better in performing a functional task.

### Comparing Different Feature Extraction and Classification Methods

**Fig 8:**
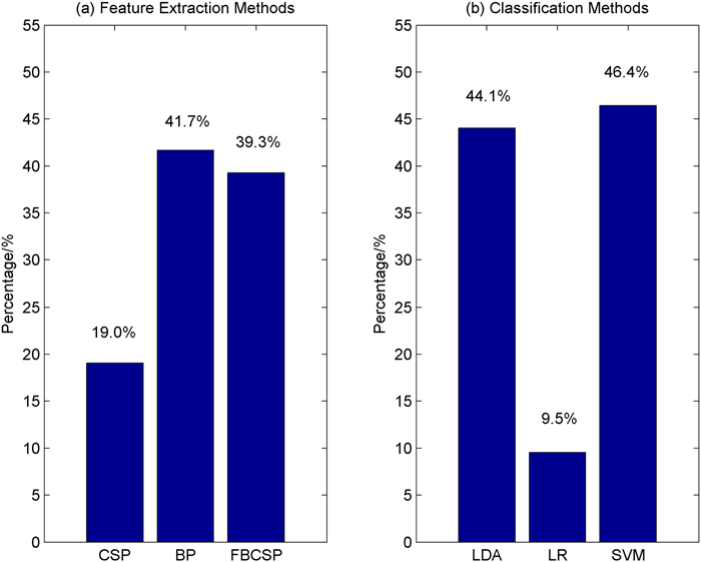
The performance of the feature extraction and classification methods. (a) The percentage of cases where a feature extraction method outperforms others; (b) The percentage of cases where a classification method outperforms others.

Nine combinations of different feature extraction and classification methods were used to discriminate the different classes of EEG signals in this study. The reported accuracy for each participant is the highest cross-validation accuracy obtained from one of the nine algorithms. We are interested to know which of the feature extraction and classification methods lead to high performance. Thus, for each of the feature extraction methods, the percentage of cases where it outperforms other feature extraction methods is computed and shown in Fig. 8 (a). Fig. 8 (b), on the other hand, shows the percentage of the number of cases where each of the classification methods outperforms other classification methods.

The feature extraction method that has the highest percentage is the logarithmic band-power method (41.7%), followed by FBCSP (39.3%) and CSP (19.0%). For the classification methods, SVM with an RBF kernel is the best classifier where 46.4% of the times, it outperforms other classifiers such as LDA (44.1%) and logistic regression (9.5%). Of all the nine combinations of the algorithms, FBCSP with SVM and BP with LDA perform the best. They respectively yield the highest cross-validation accuracy 20.2% of the times respectively. CSP and SVM, on the other hand, has a percentage of 16.7%.

**Fig 9:**
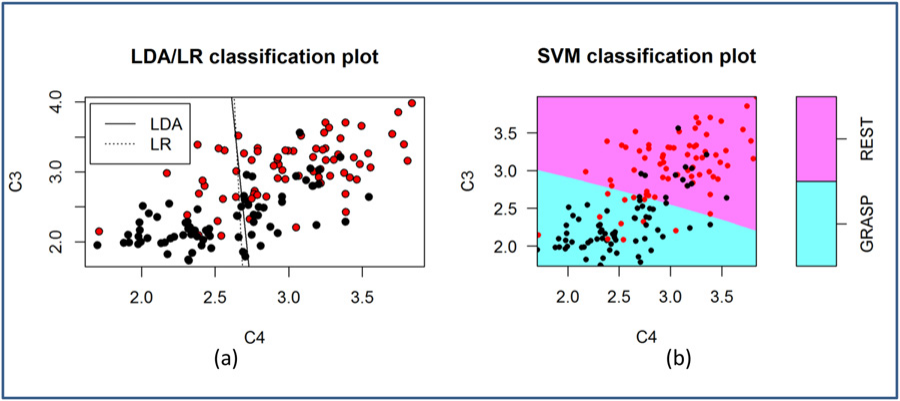
The decision boundaries of LDA, LR, and SVM when classifying REST against MI-GRASP. The red and black circles represent samples from REST and MI-GRASP respectively.

**Fig 10:**
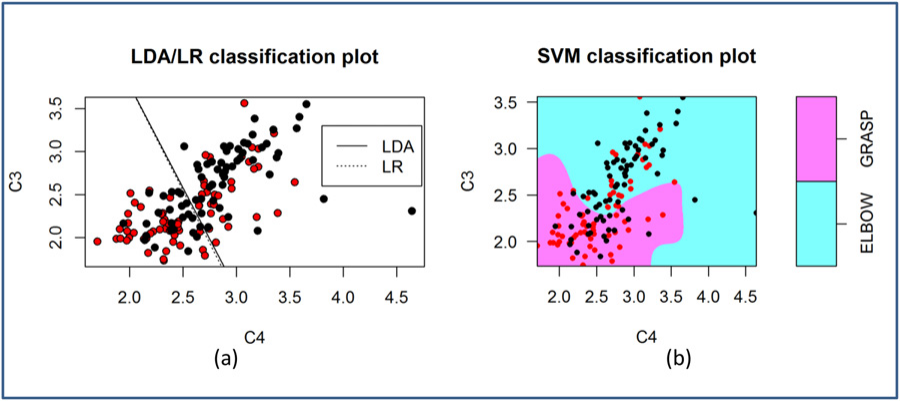
The decision boundaries of LDA, LR, and SVM when classifying MI-ELBOW against MI-GRASP. The red and black circles represent samples from MI-GRASP and MI-ELBOW respectively.

An understanding of the properties of the features is important when choosing a classifier. Even though logarithm was applied to all the features in this study, the features do not have a multivariate normal distribution as assessed by the Mardia’s multivariate normality test (*p* < 0.05) in most cases. Despite the violation of the normality assumption, LDA appears to be quite robust. 44.1% of the time, LDA outperforms LR and SVM, which makes no assumptions on the distribution of the data. Fig. 9 and Fig. 10 compare the decision boundaries of the three classification algorithms for the two-class problem Rest vs MI-GRASP and MI-GRASP vs MI-ELBOW respectively. For visualization purposes, these decision boundaries are derived using only two features: logarithmic band power at C3 and C4 using the EEG data collected from P03. The features are not normally distributed according to the Mardia’s test. For Rest vs MI-GRASP (Fig. 9), the decision boundary of SVM is almost linear. SVM, as well as both the linear classifiers (i.e., LDA and LR) produce a high accuracy of approximately 81.0% respectively. For MI-GRASP vs MI-ELBOW (Fig. 10), the classification problem becomes challenging as the data overlap more in the feature space. Thus, the classification accuracy achieved by LDA and LR is low, i.e., approximately 53.0%. The decision boundary of SVM is non-linear resulting in a higher accuracy 55.0%. As only two features were employed, the classification accuracies achieved in these two examples were smaller than those presented in Table 4 and Table 5.

**Fig 11:**
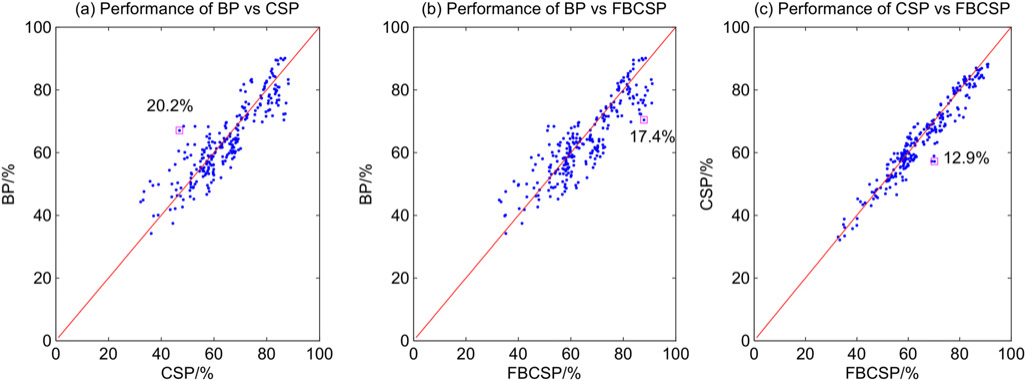
Scatter plots of the performances of different feature extraction algorithms.

**Fig 12:**
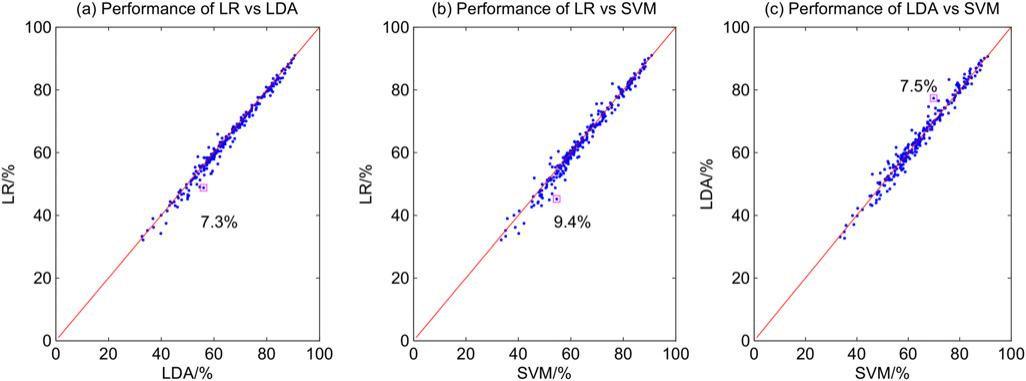
Scatter plots of the performances of different classification algorithms.

The scatter plots in Fig. 11 compare the performance of the three different feature extraction methods. The comparison between the performance of the three different classification methods, on the other hand, is illustrated in Fig. 12. The red line in each scatter plot reflects a condition where the two algorithms under consideration achieve the same accuracy. Points that deviate from the red line are instances in which one algorithm outperforms the other. The larger the deviation, the performance difference between the two algorithms is greater. As shown in Fig. 11, the differences between the feature extraction methods are pronounced. The largest differences are a) 20.2% between CSP and BP; b) 17.4% between FBCSP and BP; and c) 12.9% between FBCSP and CSP. The performance difference between the the classification methods is smaller (see Fig. 12). The largest performance difference observed between LDA and LR is 7.3%, between SVM and LR is 9.4%, and between SVM and LDA is 7.5%. As the performance difference between algorithms can be large, it is important to use appropriate feature extraction and classification algorithms to optimize the BCI performance. The choice of the features, however, affects the BCI performance more compared to the choice of the classification algorithms used in this study.

### Potential Applications

The results obtained from this study are promising. The proposed BCI can increase the number of degree of freedom of the robotic system designed in our lab for stroke rehabilitation or for assistive purposes. For example, the stroke patients can use imagine elbow movements to extend the robotic arm when reaching out for a target object (e.g. a cup), and then imagine grasp movements to activate the FES and close their fingers to grab the object. For rehabilitation purposes, the same strategy could be used to perform task-specific exercises (e.g. picking up a bean bag and place it on one of the other four locations on the table). Task-specific training refers to a therapy in which patients practice goal-oriented motor tasks they would use in daily living such as a drinking task [65]. Studies have shown that task-specific training after stroke results in better functional outcomes [66]. In addition, task-specific training has been shown to produce long lasting cortical reorganization compared to traditional stroke rehabilitation [65, 67]. In another study, Boyd *et al.* investigate if target-specific or non-specific use of the hemiparetic arm would result in functional reorganization of the contralesional motor cortex after stroke [68]. It has been reported that task-specific training plays an important role in producing plasticity in the cortex [68].

## Conclusions and Future Work

In summary, we have demonstrated in the present study that same-limb motor imagery classification is possible. For the binary classification of imaginary grasp and elbow movements, the average accuracy achieved is 66.9%. On the other hand, the performance achieved when classifying three classes of EEG signals (i.e., rest, imaginary grasp, and imaginary elbow movements) is 60.7%, which is significantly larger than the random classification of 33.3%. Our results also show that goal-oriented motor imagery leads to higher classification performance.

In our future work, the proposed three-class BCI system will be integrated with an exoskeleton robotic arm and an FES to help stroke patients in performing task-specific exercises during rehabilitation. Consequently, the efficacy of the system will be evaluated. It would also be interesting to investigate the performance gain achieved when a hybrid BCI system that combines the BCI with EMG is used to operate the rehabilitation system. This proposed system aims to promote engagement amongst stroke patient when they are undergoing rehabilitation. More specifically, the system encourages stroke patients to perform mental rehearsal of a movement (i.e., engage in motor imagery) and at the same time, attempt to generate muscle movements that match their intention to move. Subsequently, the robotic exoskeleton would provide feedback and assist the patients in performing the desired movements. We believe that such a system can potentially lead to better functional outcomes.
